# Persistent body size bias in the fossil record of Cenozoic North American mammals

**DOI:** 10.1098/rspb.2025.1780

**Published:** 2025-11-12

**Authors:** Adam Lindholm, Roger A. Close

**Affiliations:** ^1^ Department of Earth Sciences, University of Oxford, Oxford, UK

**Keywords:** body size, sampling bias, taphonomy, macroecology

## Abstract

Body size is a key organismal trait with profound implications ranging from individual physiology to large-scale macroecological or macroevolutionary phenomena. Among extant terrestrial vertebrates, peak diversity commonly occurs at small body size. Similarities between the body size distributions of fossil and extant mammals have been used to argue that fossil record signals are robust, yet preservation and collector biases disproportionately favour the sampling of large taxa and probably under-represent small-sized diversity. Here, we quantify the effects of these biases on the body size distributions of North American mammals through the Cenozoic. We assess how these distributions have changed with new palaeontological discoveries and evaluate sampling standardization as a potential correction for body size bias. Our results show bias in the mammal record to be persistent and severe. Sampling standardization has no consistent effect on recovered distribution shape probably because sample coverage estimators cannot account for changes in the scope of the sampling universe driven by a combination of historical worker interest and the preservational characteristics of a small pool of formations. Short of a novel standardization method that can account for publication biases, deriving non-artefactual fossil body size signals may ultimately depend on targeted, systematic sampling of exceptional deposits.

## 1. Introduction

Body size is one of an organism’s most important physical traits. It has direct ecological or biological (e.g. [1,2]) and evolutionary (e.g. [3,4]) effects, and its influences range from individual (e.g. basal metabolic rate) to global scales (e.g. the distribution of species with latitude). In macroecology, a key tool for understanding how body size varies across space, time and taxonomy is the body size distribution. Body size distributions are histograms of a focal group’s species diversity divided into discrete body size categories, with size typically quantified as body mass or body length, log-transformed to make relative diversity changes between the categories more similar [5–7]. Body size distributions have been computed for a wide range of extant animal groups across multiple continental assemblages, and most display a consistent geometry: pronounced positive skew (more small species than large) with a primary mode at a small but non-minimum body size (e.g. [6,8]), and frequently one or two accessory modes at larger body sizes [9–11]. There are some exceptions to this trend, notably the tendencies for distributions to display negative skews for ectothermic groups (possibly a metabolic effect [7]), to flatten and become more discontinuous with decreasing geographic scale (e.g. [12]) and to vary in skew and/or modality from a parent taxonomic grouping for its subclades (e.g. [13]). At large taxonomic and spatial scales, however, positively skewed body size distributions are broadly ubiquitous among endotherms.

In deep time, the fossil record has been used to quantify how body size distribution geometries have changed across groups’ evolutionary histories, primarily among terrestrial vertebrates. The body size distribution of Cenozoic fossil mammals, on its face, approximates its modern equivalent fairly well, with positive skew, a small-size primary mode, and a clear accessory mode at larger size [14]. The magnitude of the accessory mode in the fossil record, however, is much greater than in the modern. Part of this results from the extinction of megafaunal species at the end of the Quaternary, an effect which was strong enough in the Americas to damp the accessory mode to the point that the mammalian body size distribution was initially believed to be unimodal [9,15,16]. Nevertheless, these extinctions cannot fully explain the relative magnitude of the large-size mode: only approximately 200 species larger than 10 kg were lost in the Quaternary, yet fossil data imply that in the interval spanning the last 10 Ma, the diversity of 30–100 kg mammals was similar in magnitude to that of rodent-sized species [17] (today, rodents comprise approx. 2000 species [18]). This discrepancy results from persistent and well-documented taphonomic and collector biases against small body size in the fossil record [11,19,20]: small remains are more likely to be destroyed than large remains, to be missed or ignored in surveys and to be under-reported in the literature. This leads to a pronounced under-representation of small taxa relative to large taxa in fossil body size distributions. Aspects of this bias have been characterized for Late Cretaceous dinosaur-bearing formations in North America (Dinosaur Park and Hell Creek/Lance) [20–22] and for recent mammalian and avian death assemblages in Amboseli National Park, Kenya [19,23]. In these cases, small taxa were recovered less frequently than large taxa, and mammals showed an increasing divergence between death assemblage and living diversity with decreasing body size (presumably even greater for less than 1 kg ‘micromammals’ not examined by [19]). Small taxa were also found to preserve in much poorer quality, typically as disarticulated skeletal elements rather than the more complete skeletons of larger taxa [20,22]. Extrapolating trends from these case studies to the larger scales of previous fossil body size distributions would lead to the inference of spurious features, such as a more prominent large-size mode or even negative distributional skew [17,22]. The latter is of particular interest for dinosaurs, for which the face-value body size distribution is negatively skewed, contrasting with essentially all modern terrestrial vertebrates [14,24,25] and leading to considerable debate about whether this is artefactual or a genuine biological signal (e.g. [17,20,22,26,27]). The lack of functional or ecological correlates with dinosaurs in the modern fauna makes this debate difficult to directly resolve. This is not the case for mammals, however, and signs of substantial body size bias in such a well-studied group would suggest a much lower ability to derive accurate macroecological body size signals from fossils than previously thought. To date, though, no study has conducted a detailed assessment of the effects of body size bias across the entire Cenozoic mammalian fossil record.

Despite this, it has been argued that since the fossil mammal body size distribution retains the general shape of the modern, it is robust to the existence of these biases [14] and that legitimate conclusions can therefore be drawn about mammal body size evolution. These interpretations have largely focused on when multimodal distributions appeared in North America. In the early Cenozoic, when most mammal taxa were plantigrade [9] (though see [28]), the distribution appears to have been genuinely unimodal [10,29]. Following the 49.1 Ma [30] Early Eocene Climate Optimum, the proliferation of open habitats is thought to have enabled the diversification of large digitigrade carnivores and unguligrade herbivores, which restricted the size of most plantigrade taxa beneath approximately 0.35 kg and formed an accessory, roughly lognormal distribution centred at larger body size [9,31]. At the same time, the loss of forests is thought to have driven the disappearance of many arboreal frugivores (typically approx. 1–4 kg), reinforcing a size gap in the distribution [15,32] and leading to apparent stable bimodality from approximately 40 Ma until the Quaternary megafaunal extinctions [10]. From these findings, we *a priori* expect the mammalian body size distribution to be positively skewed throughout its evolutionary history, unimodal prior to the mid–late Eocene, and multimodal afterwards, though with the large size mode(s) of much lower diversity than the primary mode at small size. However, considerable and variable body size bias appears to impact the North American Cenozoic: figure 5.3 of [10] shows that the distribution attained negative skew at 20 Ma (Burdigalian; not discussed by that study), which could potentially cast doubt on these expectations.

Because a pervasive signal of body size bias can obscure our ability to draw reliable macroecological conclusions from fossil body size distributions, there is a clear need to directly assess, quantify and ultimately correct for it. Mammals are an excellent case study because their bias-affected fossil distributions can be directly compared with their exceedingly well-studied modern equivalent (e.g. [10,12,13,15]). Here, we use the well-sampled continental fossil mammal assemblage of North America (e.g. [33]) to quantify the impact of body size bias. We document how fossil body size distributions have changed through the history of palaeontological discovery (= ‘research time’; e.g. [34]) and characterize the maturity of sampling across different body size categories. We also evaluate the ability of coverage-based sampling standardization to control for the relative under-representation of small taxa.

## 2. Material and methods

### (a) Data download

Fossil mammal occurrences were downloaded from the Paleobiology Database (PBDB, http://www.paleobiodb.org) on 24 January 2025 using R v. 4.3.1 [35] and filtered to North America during the Cenozoic (Palaeocene–Pleistocene; see Dryad [36] for full data and code). To reduce the effects of variation in time interval duration on diversity estimates, we used equal-length time bins constructed by aggregating stage-length intervals (following e.g. [37]; see electronic supplementary material, table S3); occurrences were assigned to a bin if greater than 50% of their duration fell within it (‘majority rule’). Those not meeting this criterion were removed. Trace taxa and occurrences with a geographic scale of ‘basin,’ a stratigraphic scale of ‘group,’ or no coordinates were also removed. To limit focus to terrestrial mammals, the clades Chiroptera, Cetacea, Sirenia, Pinnipedimorpha and Desmostylia were all removed. We considered potential greater terrestrial ability in early-diverging members of marine clades, but all were primarily or wholly aquatic [38–41]. After cleaning (and applying synonymies missed by the PBDB, see electronic supplementary material, S1.1–S1.3), the dataset contained 27 356 occurrences from 3638 species. To allow us to reconstruct discovery curves from PBDB occurrence data, our primary download included all identifications. In our analyses, we also followed the procedure of [34] to drop obsolete occurrences, accounting for cases of historical specimen re-identification without a change in taxonomic opinion.

### (b) Body mass calculation and grouping

Body mass estimates were computed using log-linear allometric regressions based on skeletal measurement proxies. The two most popular mammalian mass proxies are the dimensions of long bones [42,43] and teeth [43,44]. Teeth are more commonly preserved and more diagnostic for mammals than postcranial material [43], meaning dental-based regressions would be applicable to a larger number of taxa, so these were preferred.

Many different mammalian dental-based mass estimations exist (see electronic supplementary material, S1.4), including regressions for mammals as a whole, but the majority are tailored to specific subclades. Using multiple different regressions within a single dataset necessarily imparts several different sources of error unevenly throughout it, but the alternative blanket methodology is more likely to yield less accurate mass estimates [43]. We further preferred using multiple equations given precedents for doing so in analyses of large, diverse datasets (e.g. [43,45]). The final regressions chosen are summarized in electronic supplementary material, table S4. Measurements were gathered directly from the literature or measured from published figures; the full dataset can be found on Dryad [36].

After mass calculation, taxa were grouped into order-of-magnitude mass categories. This both follows the general practice of log-transforming the size axis in body size distributions [7] and helps to account for uncertainties within or between different regression methods. Any individual mass estimate will have some degree of error around that taxon’s true average mass, but this is unlikely to exceed an order of magnitude [10,45]. Taxa for which measurements were unavailable, either owing to a lack of material or reporting in the literature, were grouped into mass categories based on the average mass of close relatives (following e.g. [20]). This allowed us to retain an additional 4.7% of occurrences. This dataset can also be found on Dryad [36].

### (c) Sampling-standardized diversity estimation

We used coverage-based sampling standardization to control for heterogeneous sampling intensity between different body size categories, such as the significant undersampling effect on small taxa believed to be produced by body size bias. In principle, this approach should recover true body-size–diversity signals from fossil data by drawing down the sample completeness (= sample coverage [46]) of better-sampled (i.e. larger) size categories to the same level as the less well-sampled (i.e. smaller) ones. Standardizing the diversity of biological assemblages by sample completeness shows how many species are seen on average in a random sample of a fixed fraction of individuals from the underlying population; this method was derived independently by Alroy [47,48] algorithmically and Chao and Jost [49] analytically. Their respective methods, shareholder quorum subsampling (SQS) and coverage based rarefaction (CBR), are essentially equivalent [34]. To date, SQS/CBR has not been used to standardize body size distributions, though there is a precedent for using it in estimating diversity between different body sizes in the Palaeogene mammal record [29]. We performed coverage standardization on body-mass-categorized incidence frequencies using the function ‘estimateD()’ from the R package ‘iNEXT’ v. 3.0.1 [50–52]. We used the resultant diversity estimates, with 95% confidence intervals, to plot sampling-standardized body size distributions per time bin, which we could then compare directly with their face-value equivalents. We performed standardization at a quorum (target coverage level) of 0.8, which is appropriate given the consistency of sample coverage for most body size categories in all time bins (see electronic supplementary material, figure S1).

### (d) Charting fossil sampling through research time

In addition to directly correcting for body size bias effects using sampling standardization, we examined how sampling has changed through the year-by-year history of published palaeontological discoveries (‘research time’) among different body size categories, using discovery curves and related data visualizations. These historical analyses reveal how our knowledge of faunas has changed as palaeontological discoveries progress, both through increased sampling intensity and expansion of the accessible ‘sampling universe’ (e.g. via coverage of new geographic space, environments, taphonomic windows, or collection methodologies such as screen-washing [34]). These curves therefore provide an additional perspective that is partially independent of statistical estimates of sample completeness, which rely on relative frequencies of rare taxa (‘singletons’ or ‘doubletons’), and partly sidestep the effects of publication biases, which may skew formal sample coverage estimates through inflation of singleton frequencies [34]. Many informative variables can be plotted against research time, as detailed in the following sections.

For all historical analyses, we examined the interval from 1840 (just prior to substantial contributions to North American mammal palaeontology by Joseph Leidy and others, e.g. [53]) to 2024. We categorized mammal body masses into the simpler framework of ‘small’ (less than 1 kg), ‘medium’ (1–30 kg) and ‘large’ (greater than 30 kg) mammals, following the general classification of [32], to permit easier visualization of the data. For comparison, we also performed all the following analyses with an order-of-magnitude system of mass categorization.

### (e) Geometry of body size distributions through research time

First, we charted changes in the skew of body size distributions through research time. Prior studies of both modern and fossil communities suggest that most of a taxonomic group’s small-size diversity is discovered later in the study of that group, causing its body size distribution to become progressively more positively skewed [21,54]. In the case of heavily size-biased fossil assemblages, this manifests as the skew becoming less negative, trending towards lognormality or a skew reversal [21,22]. For this analysis, body size distributions were generated only using the narrower, order-of-magnitude size categories instead of the coarser framework. Distribution skew was computed using the function ‘skewness()’ from the R package ‘moments’ v. 0.14.1 [55]. As an extension of these concepts (see [56]), we also plotted the central tendency of mass for all discovered mammals through research time (necessarily excluding the 676 unmeasurable species), both for each geological time bin and for the Cenozoic overall. Median mass was preferred over mean mass as it is a more accurate measure of central tendency for asymmetric distributions [57]. We used only presently accepted taxon designations for these analyses to ensure skew and median mass values would be consistent with those for the body size distributions. In addition, we recorded the modal mass categories of each fossil distribution and tabulated the relative size of the large-sized mode (where present) to the small-sized mode, for further comparison with the distributions of modern mammals in [10,58].

### (f) Sample coverage through research time

Second, we examined how sample coverage changed in each of the body size categories through research time for each time interval. Coverage was estimated using Chao and Shen’s ‘improved Good’s *u* estimator’ [59] (see also [49]), which utilizes frequencies of both singletons and doubletons and has a smaller mean-squared error than the original Good’s *u* equation. Confidence intervals for the coverage estimates were calculated by bootstrapping using the package ‘boot’ v. 1.3-31 [60,61], in order to quantify the error associated with estimates derived from small sample sizes.

### (g) Discovery curve analyses

Third, we plotted discovery curves of size-categorized mammals for each geologic time bin. Through research time, discovery curves will eventually asymptote as a more complete record of species within a time interval or formation is assembled and the rate at which new species are described in the published literature diminishes [20,22]. If the publication of fossil occurrences reflected random sampling of the fossil record and the size of the sampling universe were static, then discovery curves would agree with formal sample coverage estimators. However, differences could arise if publication biases cause reporting to depart from this idealized scenario [34]. This can be used to identify how mature our knowledge of the fossil records of different body size categories is—small taxa might be more likely to show a continued increase in diversity towards present day, while the curves for larger taxa might asymptote.

To account for the significant effect that changing taxonomic opinions can have on estimates of species diversity through time (e.g. [22,56]), we performed analyses on the identified name that was current for each year of research time for each PBDB occurrence in the dataset. Two possible reasons for changes in identified names were considered: (i) recombination (species retained as valid but assigned to a different genus) and (ii) synonymization (species considered invalid and grouped into another taxon). Changes of type (i) were tracked using recombination data in a PBDB download of taxonomic opinions and type (ii) were tracked using recorded synonymy information on the PBDB website (available on Dryad [36]). For each year of research time from 1840 to 2024, entries were renamed according to these changes before the number of discovered species was computed.

We also constructed sampling-standardized discovery curves (SSDCs) for each size category and time bin, following [34]. When standardized to equal sample coverage, diversity through research time should remain flat unless the scope of the underlying sampling universe expands, or if publication biases favour the description of novel taxa over reporting new occurrences of known taxa. For the SSDCs, we performed sampling standardization as outlined above at a quorum of 0.8. For each time interval, a given size category was not considered viable for standardization until it contained three occurrences because of the high error associated with coverage estimation at very small sample sizes.

We plotted all discovery curves against two different temporal axes, one using the cumulative number of chronologically ordered occurrences, and the other being a more conventional plot against publication year. Occurrence-ordered curves, following [34], reveal when apparent discovery asymptotes through research time in the ‘traditional’ curves are driven by a lack of recent collection or publication effort rather than higher sample completeness. We also plotted an additional (non-standardized) discovery curve against the cumulative number of chronologically ordered references (again following [34]), which can more clearly emphasize publication and collector biases towards certain size categories, reflected by those categories having much longer curves.

We also charted sampled geographic area (as summed minimum spanning tree length) and counts of collections, sampled formations, and references through research time as additional proxies for collector effort and the scope of the sampling universe. These are discussed in electronic supplementary material, S1.7.

## 3. Results

### (a) Fossil body size distribution geometry

**Figure 1.**
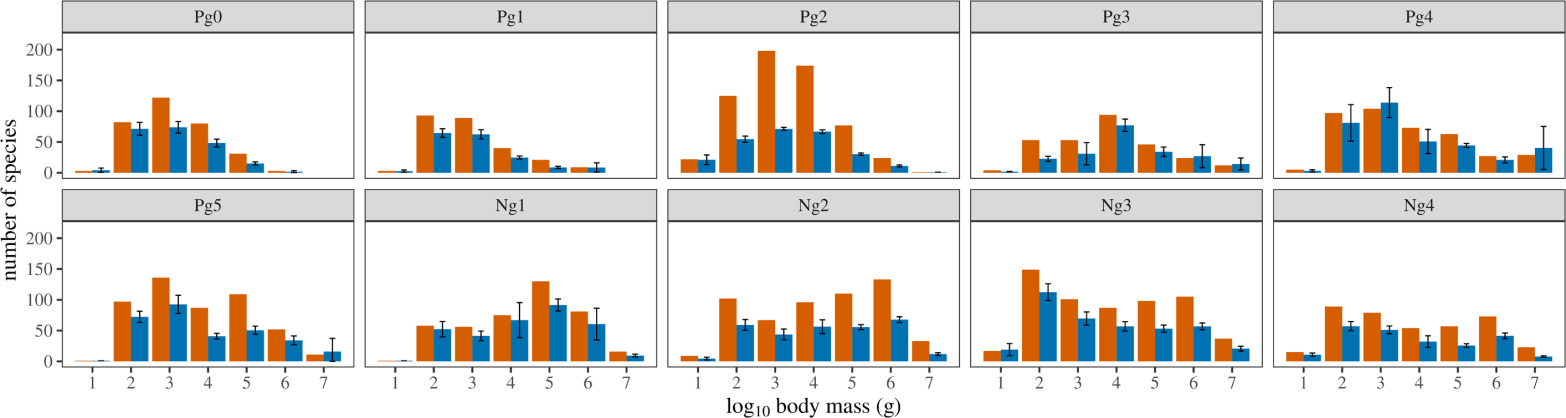
Face-value (left columns, in red) and sampling-standardized (right columns, in blue, with 95% confidence intervals) body size distributions for Cenozoic North American mammals. For Pg4-aged mammals less than 0.01 kg, standardized diversity was an extrapolation above double the reference sample size and may be unreliable.

**Figure 2.**
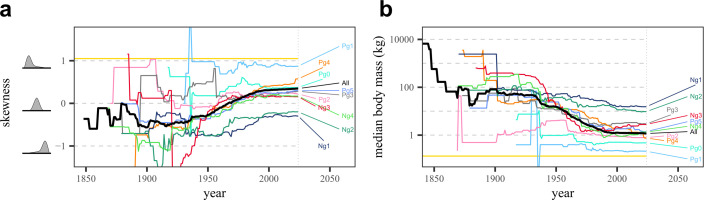
Plots of (a) body size distribution skew and (b) median species body mass for each time bin and the entire Cenozoic through historical research time. The solid horizontal line in each panel represents the Pleistocene-inclusive modern value (see electronic supplementary material, table S5). Schematic diagrams to the left of (a) are representative positively skewing, lognormal and negatively skewing distributions, for reference. Note that (b) is based on a slightly smaller taxon pool than (a), as calculating median mass must necessarily exclude taxa which could only be mass binned due to a lack of measured material.

Face-value (= unstandardized) body size distributions display an inconsistent, highly variable geometry across the Cenozoic (see figure 1). Early Neogene (bins Ng1–2; Aquitanian–Tortonian) distributions notably have negative skews, consistent with the 20 Ma body size distribution of [10], but substantially at odds with our expectation of biologically plausible distributions. Skew trends through research time have a greater degree of consistency. Generally, these show an initial period of sharp skew decrease, followed by a longer period of increase and, in most cases, an eventual shift from negative to positive skew by the present (see figure 2a). This suggests that the body size distribution in each interval has indeed become more completely sampled through research time. The change from decrease to increase occurs in the early twentieth century for all bins, roughly between the years 1925 and 1950, approximately coinciding with the development and popularization of screen-washing as a collection technique by Hibbard and others, which greatly expanded recovered small-sized mammal diversity [56] (see also electronic supplementary material, figure S2). The pattern of skew increase and reversal is most clearly seen in bins Pg4–5 (Bartonian–Chattian) and Ng3–4 (Messinian–Late Pleistocene) and to a lesser extent in bins Pg2–4 (Ypresian–Priabonian), which have remained roughly positive in skew since 1840, except for brief periods in the early twentieth century. Bins Ng1–2 show the same general trend shape as the other intervals, but never attain positive skew, reminiscent of skew trends for dinosaur assemblages in Late Cretaceous North America [21,22]. Most bins further show that skew has roughly asymptoted since approximately 2000, meaning that new species discoveries in the last 25 years have not significantly altered distribution geometry and/or that their rate has significantly slowed.

**Figure 3.**
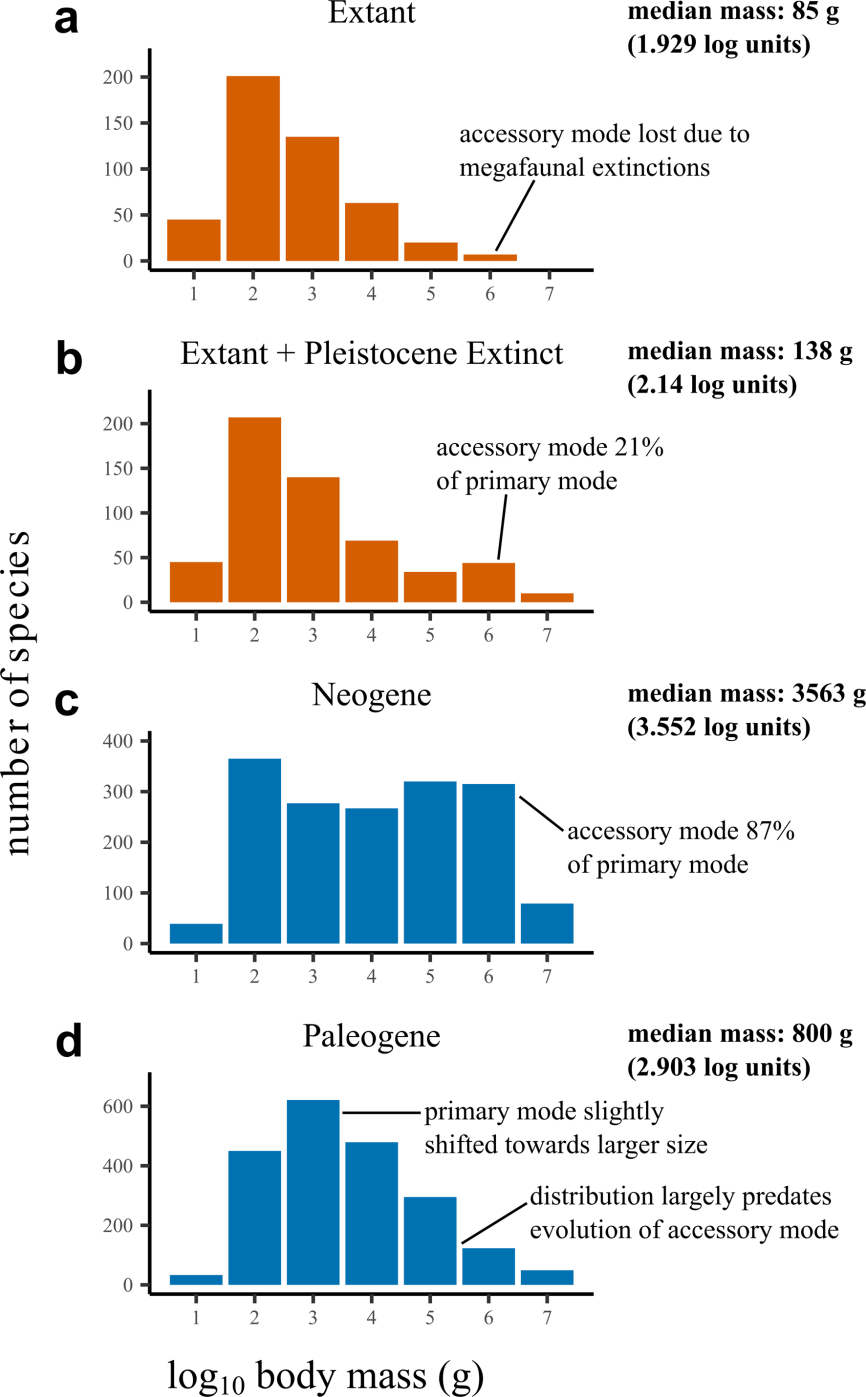
Summary body size distributions highlighting the extent of body size bias in the mammalian fossil record. (a) is the distribution of extant taxa only, (b) is the distribution of extant North American mammals along with extinct Pleistocene taxa (including megafauna), and (c) and (d) are the distributions for all fossil North American Neogene and Palaeogene taxa, respectively. Body mass data for (a) and (b) from [58].

Plots of median mass through research time reinforce these trends, with the value having decreased towards the present in all cases (see figure 2b). As with skew, these curves have broadly asymptoted in all bins, with the whole-Cenozoic curve reaching a value of approximately 1.2 kg. This contrasts with both the modern distribution, where the median mass of North American mammals is approximately 0.08 kg, and the ‘Late Pleistocene’ (modern+Pleistocene-extinct or megafaunal-inclusive) distribution, where the median mass is approximately 0.13 kg [10], both roughly an order of magnitude lower (see also figure 3 and electronic supplementary material, table S5). This contrast is further accentuated by bin Ng4, which includes Pleistocene megafauna as well as multiple extant taxa known from fossils, reaching a median mass of approximately 1.3 kg—demonstrating that the absence of giant taxa in the modern is not the primary driver of these signals, and instead suggesting that small mammal diversity throughout the Cenozoic is still considerably undersampled. The other time bins reinforce this dichotomy: all Neogene examples have median masses at least an order of magnitude larger than the modern + Pleistocene value, and the only bin that roughly approximates it is Pg1 (0.212 kg), which pre-dates the later-Eocene diversification of large mammals entirely [9,31].

The only trend in the face-value body size distributions which is fully consistent with expectations of mammalian body size evolution is the appearance of the accessory mode at large size in bins Pg3–4 (Lutetian–Priabonian). This interval begins approximately 48 Ma and is therefore concordant with the prediction that large sizes diversified in the wake of the Early Eocene Climate Optimum discussed in the Introduction. Further to this, Pg3 sees the first proliferation of taxa in the 1000+ kg size category; no 1000+ kg mammals are known in bins Pg0–Pg1 (Danian–Thanetian), and only one species in Pg2. However, the relative diversity of this large-size mode is highly variable and non-reflective of the modern distribution (necessarily Pleistocene-inclusive, as the extant-only distribution has lost enough of this mode to appear unimodal [9]). In the modern case, the large-size mode has approximately 21% the diversity of the primary, small-size mode, while for Pg3–Ng4, it is generally approximately 60–80% of small-size diversity, and up to approximately 225% including negatively skewed Ng1–2 (see electronic supplementary material, table S5).

### (b) Sampling-standardized body size distributions

Contrary to expectations, sampling standardization does not consistently draw down the relative frequency of large taxa compared with small taxa, even when the face-value distributions suggest that small taxa are substantially under-represented (see figure 1). Calculations of distribution skew between the standardized and unstandardized distributions (see electronic supplementary material, table S5) show that while standardization increased the value of skew in most intervals (Pg0–1, Pg4–5 and Ng3–4), in others it caused skew to decrease (Pg2–Pg3), which is not expected under the assumption that small taxa are under-represented in any given fossil assemblage. In negative-skewing Ng1–2, sampling standardization caused Ng2 to become roughly lognormal, but Ng1 remained strongly negatively skewed. It is highly unlikely that these negative or lognormal ‘corrected’ skews represent a legitimate biological signal given that they differ dramatically from both the extant-only and Pleistocene-inclusive modern distributions (see figure 3 and electronic supplementary material, table S5).

### (c) Sample coverage through research time

Plots of sample coverage through research time (see electronic supplementary material, figure S3) further suggest difficulties in detecting or correcting body size bias through sampling standardization. Across size categories and time intervals, Good’s *u* converges to relatively consistent values (approx. 0.8 or higher, a value largely robust to using either the simple or order-of-magnitude mass binning schemes; see electronic supplementary material, figure S13), implying that levels of sample completeness for small and large taxa are generally similar through the Cenozoic. This is at odds with the results discussed above—for example, in Ng1–2, we might expect that the strong negative skew of the face-value distributions would be associated with much lower coverage at small body sizes, but this is not the case, especially for Ng2 (where minimum coverage is 0.886, for 0.1–1 kg mammals). Furthermore, the lowest levels of sample coverage occur for 1000+ kg mammals (see electronic supplementary material, figure S13) in bins Pg3 (0.654), Pg4 (0.736) and Pg5 (0.744), deviating from the expectation that larger body size should be associated with higher overall coverage. The discrepancy between the high apparent coverage and low representation of smaller taxa may be due to complex sampling bias effects (see §4 and electronic supplementary material, S1.8).

### (d) Discovery curve analyses

**Figure 4.**
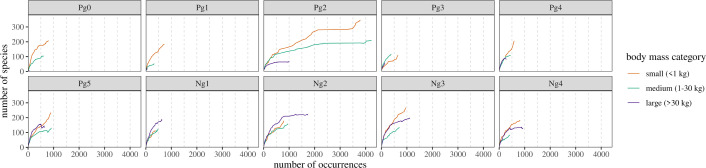
Discovery curves of species count versus chronologically ordered numbers of occurrences for North American mammals across the Cenozoic. Generally, for all time bins and size categories, curves show little indication of asymptoting, suggesting highly incomplete sampling. Bin Pg2 is a notable exception to this trend, due to the influence of the well-sampled and preservationally strong Willwood Formation.

Discovery curves plotted against chronologically ordered counts of occurrences reveal significant differences in historical research effort across time intervals and body size. Most bins show similar, sharp increases in species discovery rates across all size categories, implying that the majority of size categories remain incompletely sampled (see figure 4). There are two clear exceptions to this: large taxa in Ng2 and all size categories in Pg2, which have roughly asymptoted at higher numbers of occurrences (although there are recent increases in small/medium species diversity in Pg2). In both cases, this means that (i) these intervals have been more intensely collected than the others (perhaps for large taxa alone in Ng2); and (ii) new reports of occurrences are generally not producing novel species—i.e. the faunal record is more complete.

Unstandardized time-ordered discovery curves (see electronic supplementary material, figure S4) similarly indicate variability in the historical maturity of sampling between body size categories and time intervals, but show more frequent asymptotes among large-size classes, in contrast to the curves plotted against occurrences. These results highlight a potential shortcoming with the ‘traditional’ time-ordered methodology, where a lack of work across an interval registers as more complete sampling within it. There are minor discrepancies in the incidence of asymptotes in these discovery curves compared with a ‘description curve’ tracking only the oldest described occurrences of each species (see electronic supplementary material, figure S8). This primarily results from a PBDB ‘placeholder’ reference for North American mammal occurrences; see electronic supplementary material, S1.5.

Regardless of these effects, inconsistencies in the ranked order of diversity of the different size categories between different time bins, reflective of volatile distribution geometry (large taxa are the most diverse by the present for negatively skewed Ng1–2, while small taxa are in positively skewed Ng3–4) accentuates the variable maturity of body size sampling in both the time-ordered and occurrence-ordered curves. The only consistent trend in curve rank order is that large taxa become more diverse than medium taxa roughly between bins Pg4 and Pg5. Though the base of Pg5 occurs approximately 10 Myr after the Early Eocene Climate Optimum, this pattern is broadly consistent with the appearance of the ‘intermediate size gap’ [32] in the late Eocene. Against counts of references, most bins again show relatively steady increases in species diversity across body size (see electronic supplementary material, figure S5). However, the base of underpinning literature is clearly much greater for Neogene large taxa; Ng2 and Ng4 have roughly asymptoted large-size curves, indicating either a lack of recent research interest or near-complete description of the large fauna. All size categories have a relatively large base of literature in bin Pg2.

SSDCs, both time-ordered (see electronic supplementary material, figure S6) and occurrence-ordered (see electronic supplementary material, figure S7) display largely similar trends to their respective unstandardized equivalents; see electronic supplementary material, S1.6. Results of the analyses of spatial coverage, collections, sampled formations and references through research time also generally support the results of the discovery curve analyses. Neogene large taxa show progressively higher levels of spatial coverage and higher counts of collections and publications than small taxa; see electronic supplementary material, S1.7 and figures S9–S12. Order-of-magnitude-binned versions of all results plots are presented in electronic supplementary material, figures S13–S23.

## 4. Discussion

We quantified biases in the body size distributions of Cenozoic North American fossil mammals. Trends of median species mass through research time suggest that a significant amount of fossil small mammal diversity is missing from the record (see figure 2b), but face-value body size distributions show the relative proportion of this missing diversity is variable and that distributional geometry is highly volatile. We also evaluated whether coverage-based sampling standardization could correct for these biases, finding it inconsistent in its ability to draw down the diversity of large taxa relative to small taxa. This appears to be due to a mismatch between the completeness of sampling as detected by sample coverage estimators and by discovery curves or other proxies for sampling maturity. Estimates of sample coverage are relatively high and comparable in value for the majority of body size categories in all time bins. Discovery curves, meanwhile, indicate great variability in the completeness of our knowledge of diversity through time and between different body size categories, with larger taxa tending towards more complete or ‘mature’ sampling compared with smaller ones. This variability also extends to SSDCs—the general lack of asymptotes among these (see electronic supplementary material, S1.6) further lowers the confidence that sampling-standardized body size distributions are biologically informative. Our results are therefore consistent with past research indicating that body size biases cause small taxa to be systematically and severely under-represented in the fossil record [11,19,20,22], but our findings of the persistence and complex variability of this bias in the mammal record directly contradict prior claims that the fossil mammal body size distribution is time-stable or bias-robust [10,14].

These complex bias effects prevent the underlying occurrences in a given interval from being a true random draw of available species by changing the accessible sampling pool from which those species can be drawn and therefore skewing what is detected by sample coverage estimators towards misleading values. As with the major drivers of body size bias discussed in the Introduction, these effects can be roughly divided into two sources: taphonomy and worker interest. Taphonomic effects directly control the availability of preserved size categories through the presence or absence of certain depositional styles, which can dominate a particular time interval due to the influence of individual well-sampled formations. Worker effects (beyond more frequent sampling of specific taphonomic suites) may concentrate recovered diversity on particular local areas or taxonomic groups, employ collection methodologies which favour particular size classes, and/or govern how taxa are ultimately reported in publications. Both these effects control the scope and magnitude of recovered body size categories, and therefore change the scope of the sampling universe through the Cenozoic in a complex manner.

### (a) The influence of individual formations on observed body size trends

Trajectories of collection counts through research time suggest the amount of historical collector effort is highly uneven between different time bins. Pg2 is the most striking example, accounting for approximately one third of all occurrences in the dataset (9000+), and otherwise notable for its consistently high and broadly asymptoted discovery curves for all size classes (see figure 4). However, Pg2 is not associated with a substantially greater amount of spatial coverage or a greater number of constituent formations than the rest of the Palaeogene (see electronic supplementary material, figures S9 and S11); indeed, over half of Pg2 occurrences are derived from a single formation, the Willwood. Similar effects are seen in the other, less well-sampled time intervals, with the majority of species diversity in each tied to a handful of deposits. Therefore, the taphonomic and sedimentological characteristics peculiar to these productive formations could exert outsized control on the continental body size distributions of their respective bins.

Broadly, formation-specific taphonomic effects can be divided into three categories: (i) those favouring the preservation of small taxa, (ii) those favouring the preservation of large taxa, and (iii) the taphonomic and sampling ‘special case’ of the Willwood Formation. Only the last of these is discussed in detail here; for (i) and (ii), see electronic supplementary material, S1.8. The Willwood Formation contributes a very large number of occurrences (approx. 17%) to the dataset and has a long history of systematic fossil sampling, estimated to be approximately 50 000 specimens from over 1000 individual localities [62,63]. Body size sampling varies within the Willwood due to differences in hydraulic size-sorting of remains between palaeosol facies. This means a significant taphonomic control exists over locality-scale body size distribution geometry [62,63]. However, several well-collected localities (i.e. Nowater Creek) have yielded highly diverse small faunas which include rare clades [63], and the formation-scale body size distributions remain broadly consistent across the entire Willwood succession [64], so the formation average may represent a reliable signal (though this will incorporate some degree of species-area turnover [63]; see also §4(b)). Furthermore, the reported species diversity of the Willwood is roughly equivalent to that of the contemporaneous Wasatch Formation, which has five times fewer occurrences, suggesting the Willwood species record could be reasonably complete (see also [65]). The Willwood Formation also alleviates some concerns regarding low large-taxon diversity in the Early Palaeogene (see electronic supplementary material, S1.8): large mammals are well-sampled from Willwood mudstones [64], meaning their lower diversity is probably a genuine signal. Body size biases are still present in Willwood and broader Pg2 material (e.g. the modal body size of Pg2-aged mammals is an order of magnitude higher than the present; see electronic supplementary material, table S5), and the rarity of large mammals is probably underplaying the strength of this effect. Regardless, the collective preservational fidelity and systematic sampling history of Willwood material mean Pg2 data are more likely to be biologically informative compared with other intervals (see electronic supplementary material, figure S24) and perhaps represent maximally thorough faunal sampling across the range of body size at the scale of an entire unit.

### (b) Worker effects on body size trends through space, sampling methodology and publication

The fidelity of Willwood Formation body size signals is not solely due to its preservational conditions but also to its history of thorough, systematic fossil collection by several institutions [63,65]. Indeed, the taphonomic characteristics of any major occurrence-contributing formation only have a strong effect on the shape of the body size distribution because of worker interest: collector-driven body size bias is a pervasive influence on fossil body size data. Outside of simply targeting formations to increase their relative contributions of fossil occurrences, worker effort effects can be broken down into three main categories: (i) sampled spatial area, (ii) sampling methodology and (iii) publication.

One consequence of the dependence of the North American mammalian fossil record on a small number of formations is that the spatial coverage of a given interval may be dominated by a small geographic area. This could have a substantial impact on the recovered body size distribution: the limited area of focus would miss changes in faunal composition resulting from spatial turnover of species’ identities, which in modern mammals is greatest among modal-sized taxa (both large taxa tend to have larger ranges) [8,12]. This effect is perhaps strongest in the late Palaeogene and early Neogene, where most occurrences derive from a relatively small area in Nebraska (to a lesser extent for the former; the Pg4–Pg5-aged White River Group covers a wider extent in the American Western Interior [66]). In addition to taphonomic biases in Ng1–Ng2-aged formations which greatly limit the observed diversity of small mammals (see electronic supplementary material, S1.8), the small sampling area probably contributes to the negative distributional skew in these intervals (and could cause similar problems for Willwood data, though it has been noted Willwood deposits span a range of different habitats and communities [63]).

Into the later Neogene, spatial coverage increases dramatically, especially for large taxa (see electronic supplementary material, S1.7). The discrepancy between the underlying spatial sampling pool for large and small taxa could further explain the under-representation of small size diversity, since it would cause undersampling of spatial turnover for small-bodied species as discussed above. However, this could also be due to historical preferences for particular sampling methodologies, which is perhaps the most pronounced form of collector bias. As discussed in electronic supplementary material, S1.8, Ng3–Ng4 sees greater availabilities of fluvial/predatory/cave microsites and unlithified deposits, both of which can greatly increase known small size fossil diversity [67]. Although screen-washing unlithified deposits became an established practice for collecting small fossils from the 1930s [56], it is not always performed, and the alternative strategy of surface prospecting will instead skew recovered specimens towards large size [11,68–70], an effect also seen in surveys of modern death assemblages [19,70]. More frequent implementation of surface prospecting in late Neogene deposits could directly explain the much greater spatial coverage of large taxa in the associated time bins (see [11] regarding late Neogene Eurasia). It is also worth noting that not all surface surveys are so strongly size-biased. Much of the Willwood Formation was intentionally sampled by surface prospecting instead of screen-washing, and localities like Nowater Creek apparently had sufficient preservational fidelity that the prospected assemblage resembled those screen-washed or quarried from other formations [63]. An intensive survey of similarly exceptional localities in other formations could therefore be beneficial for yielding more accurate fossil body size data.

The final major worker effort bias is in publication, with historical interest in particular groups ballooning their diversity and associated occurrence count, and therefore greatly increasing their apparent sampling maturity relative to other (usually small) taxa. This is perhaps best exemplified by the large number of systematic reviews focusing on medium or large-bodied groups, such as camelids [71], oreodonts [72,73], rhinoceroses [74], canids [75,76] and peccaries [77], which typically include fairly exhaustive specimen lists for all species examined. This could explain why early Neogene discovery curves against occurrences and references show rough asymptotes for large taxa while reference counts continue to increase (though slowing in the 2010s–2020s): large taxa have attained a very mature reference pool. Small taxa, meanwhile, generally suffer from a much less comprehensive reporting of occurrences, and even if recent collections of relevant material have become more thorough, could be missing considerable quantities of unpublished, unreported ‘dark data’. Publication biases are further likely to inflate the occurrence pools of large taxa through simple desire to focus research on more ‘charismatic’ species, similar to early interest in collecting the very largest specimens for museum display in the dinosaur fossil record [24] (though the aforementioned improved collection methodologies have reduced this particular effect).

Greater worker interest in particular size classes not only inflates associated reference and occurrence pools but also causes taxonomic inflation. This is best explained by two discovery trends in Pg4 and Pg5. In the former case, small-size diversity spikes in the 2020s due to the publication of a monograph describing 10 novel species collected from harvester ant mounds in Nebraska ([78]; a signal further inflated by the artefactual reference discussed in electronic supplementary material, S1.5, though as noted in that section, this does not alter our interpretations). Conversely, in Pg5, medium and large species show a marked decrease in their known diversity due to a series of mid-1990s taxonomic revisions of oreodonts [72,73] that reduced previous spikes driven by mid-twentieth-century monographs by Schultz and Falkenbach (e.g. [79]) through a large number of synonymizations. Both these effects highlight the relative balance between taxonomic ‘lumping’ and ‘splitting’ that can greatly shift fossil diversity estimates (e.g. [22]). Among large Cenozoic mammals, these competing interpretations have tended to reach a point of stasis (though not always—varying interpretations of the taxonomy of derived brontotheriids advocate either 2 or nearly 30 species [80,81], with the PBDB favouring the much older ‘split’ interpretation), but small mammals are probably subject to some degree of taxonomic inflation due to a lack of historical time for revision in the face of accelerated discovery [56]. This results in a complex bias signal: small taxa are preserved and/or discovered less frequently than large taxa but also may require their estimated diversity to be reduced to account for unsettled taxonomy. Taxonomic inflation effects are also closely tied to the ‘new species of X from Y’ problem, which sees species diversity (and counts of singletons) progressively rise due to literature focus on describing new species—a signal which is robust to all forms of subsampling [82].

Ultimately, worker effort biases are complex and serve to alter the size of the accessible sampling universe in different ways. Variation in sampled spatial area between bins means certain intervals are missing larger proportions of the continental fauna than others, even if the sampled formations could be considered roughly sample-complete (though recall these are still susceptible to taphonomic biases). Lack of systematic sampling of small taxa through screen-washing or focused collection of microsites creates a mismatch between the apparent coverage and diversity of large and small taxa. Finally, worker interest in large-bodied groups and variability in how ‘settled’ taxonomy is between groups of differing body size can further shift the sampling pool away from being a random draw of the fossil fauna in any given interval, again causing completeness as calculated by sample coverage not to reflect completeness as indicated by rates of taxon discovery.

## 5. Conclusions and prospectus for body size distributions in the fossil record

Although it has been claimed the fossil mammal body size distribution bears a strong resemblance to its modern equivalent (at least pooled for the entire Cenozoic) [14] and that this distribution is time-stable in its basic geometry [10], our analyses instead show the distribution to be highly volatile through time. Different time intervals show variable underestimation of diversity at small body sizes, but this underestimation is always substantial—a fact underscored by the enormous differences in distributional geometry between all observed fossil assemblages and the modern, even when Pleistocene-extinct megafauna are included in the latter (see figure 3). Distributional skew also varies between different time intervals, with some having pronouncedly negative skews. This supports the idea that the face-value shape of fossil body size distributions can be an artefact of body size biases (e.g. [17]). Negative skews in mammalian distributions further suggest particular caution should be taken in explaining similar patterns in other fossil groups (i.e. dinosaurs) as the result of unique life history or ecology, because these do not apply to mammals. Variation in body size bias necessarily explains most of the variation in distributional geometry across the Cenozoic, itself governed by a complex interplay of historical worker interest and the taphonomic characteristics of individual fossil-bearing formations. Despite biases, some aspects of mammalian body size evolution predicted by previous studies are still recorded by their body size distributions. An accessory mode at large sizes appears in the mid–late Palaeogene (meaning the distribution shifts from unimodal to bimodal [9,10,31]), and large taxa become more diverse than medium-sized taxa on a similar time frame, in line with the appearance of the ‘intermediate gap’ in the distribution [15,31,32].

Coverage-based sampling standardization showed little consistency as a strategy to correct this bias despite high apparent sample coverage in all size classes and time intervals; body size bias probably has effects on distributional shape beyond the prevalence of singletons across different size categories. A stronger alternative to correcting body size bias may be to focus the study on exceptionally preserved or sampled deposits which could yield more accurate data on diversity across body sizes. The primary such exemplar identified by this study is the Pg2-aged Willwood Formation, which has been thoroughly sampled due to a long history of systematic fossil collection with the intention of ‘exhaustively’ expanding its species inventory across mammalian taxonomy [63]. Its significant contribution to the diversity of Pg2, diversity which has broadly asymptoted for all size categories and has a much larger underlying occurrence pool than any other bin (see figure 4), probably renders body size signals therein more accurate, or at least accurate at a regional geographic scale, given that the limits of formation size probably miss aspects of faunal change associated with spatial turnover. Furthermore, in the wider geological record, the Willwood may represent a similar preservational or collector case to well-studied Konservat Lagerstätten such as the early Cretaceous Jehol Biota of China, which has been argued to yield more accurate body size-diversity data for dinosaurs [17]. More recent geological intervals (those post-dating the appearance of the accessory digitigrade-unguligrade body size mode) that contain productive microsites (*sensu* [67], though see electronic supplementary material, S1.8) or have histories of screen-washing surveys or other systematic sampling expeditions also have the potential to yield more accurate data.

It is the discovery, collection and description of novel small species that drives the progressive increase in the skew of body size distributions among both fossil [21,22] and extant [54] animal groups. In the fossil case, our ability to assess the completeness of species records across body size categories is obscured by formation-specific taphonomic effects and worker interest effects. These limit the relative scope of the sampling universe between size categories and tend to favour larger taxa. Our results show that in the North American Cenozoic mammal record, these biases have a much stronger impact than previously suggested, though not to the degree that non-artefactual body size data is rendered unobtainable. The best way to obtain more accurate body size data in the future may come from (i) further modelling and examination of the existing record in order to disentangle worker and taphonomic effects—thereby potentially simplifying the body size distribution to one affected by taphonomic biases alone and enabling more direct comparisons with the modern; and (ii) the continued systematic, thorough collection and publication of material from exceptional fossil deposits.

## Data Availability

Supplementary text, figures, and tables are available in the supplementary material [83]. Mammal measurement data and all analysis code can be found on Dryad [36].
